# Effect of Decontamination and Cleaning on the Shear Bond Strength of High Translucency Zirconia

**DOI:** 10.3390/dj5040032

**Published:** 2017-11-14

**Authors:** Stephanie Krifka, Verena Preis, Martin Rosentritt

**Affiliations:** Department of Prosthetic Dentistry, University Hospital Regensburg, 93051 Regensburg, Germany; verena.preis@ukr.de (V.P.); martin.rosentritt@ukr.de (M.R.)

**Keywords:** high translucency zirconia, saliva contamination, bond strength, surface cleaning, decontamination

## Abstract

(1) Background: This study evaluated the bonding performance of high translucency zirconia after diverse surficial decontamination and cleaning procedures. (2) Methods: High translucency zirconia (Lava^TM^ Esthetic) specimens (2.0 mm × 20 mm × 10 mm) were exposed to different surface treatments prior to bonding to CoCr cylinders (d = 5 mm, height = 3 mm). All surfaces were sandblasted (40 µm aluminum oxide, 2 bar) and treated with alcohol (al), saliva (s), saliva + water (sw), or saliva + NaOCl + water (sn) before bonding was performed with the following adhesive luting systems: RelyX^TM^ Unicem 2 (RX), Scotchbond^TM^ Universal (SBU) + RelyX^TM^ Ultimate (RU) or Monobond Plus (MP) + Multilink^®^ Automix (ML). After 24 h, thermocycling (TC:12,000 × 5 °C/55 °C) and 90 days of storage at 37 °C in distilled water, the shear bond strength (SBS) was evaluated according to ISO/TS 11,405:2015. Failure modes along bonding areas were characterized. Means and standard deviations (*n* = 10 per group) were determined and statistically analyzed with one-way ANOVA/Bonferroni (α = 0.05). (3) Results: The SBS after 24 h varied between 3.5 (sRX) and 69.4 MPa (snMP + ML). Values from 0 (sRX) to 70.3 MPa (swRX) were found after TC. Data after 90 days of storage showed the lowest values for sRX (0 MPa) and the highest values for alSBU + RU (75.5 MPa). Adhesive failure was noted at all aging conditions. (4) Conclusions: SBU + RU or RX and MP + ML including saliva decontamination of the ceramic surface with water or NaOCl + water allow efficient bonding to Lava^TM^ Esthetic.

## 1. Introduction

Dental restorative materials for single crowns and fixed or partially fixed dentures are intended to mimic the visual nature and function of the original teeth. Silica-based ceramics, e.g., feldspathic (flexural strength ≤ 160 MPa) or lithium disilicate ceramic (≤400 MPa), are predominately utilized for single tooth restorations. Non-silica-based or oxide ceramics, such as high-strength yttria-stabilized tetragonal zirconia polycrystals (3Y-TZP; ≤1200 MPa), are increasingly being used as veneer frameworks or monolithic material to restore and rehabilitate partially edentulous patients. The inferior translucency of standard monolithic zirconia restorations adversely affects their clinical application. Aesthetic demand for high translucency zirconia produced by computer aided design and computer aided manufacturing (CAD/CAM) for monolithic restorations has increased. Improved translucency results from changes in zirconia structure due to an increase in yttria content from 3 to >5 mol %. The tetragonal zirconia phase decreases to more cubic phase particles, causing a reduction in flexural strength (600–800 MPa) [1]. In addition to the biological, chemical, and physical properties of the material, the clinical performance of all-ceramic restorations is significantly influenced by adequate adhesive bonding after the necessary preparations of the tooth and ceramic surfaces [1,2,3]. Ceramics with a high glass content (silica-based ceramics) are hydrofluoric acid (HF) etchable followed by silanization [4,5] and adhesion of resin luting systems is achieved by micromechanical interlocking and chemical bonds. Since oxide ceramics are inert to HF, the ceramic surface is roughened either by soft (50 µm, 1 bar) airborne-particle abrasion followed by multifunctional primers with adhesive phosphate monomers or silicatization combined with silanization (tribochemical treatment) to form a durable bond with the resin cement used [2,3,6,7,8]. Ceramic restorations are tried in and fitted to abutment teeth before cementation. During this process, the restoration surface is contaminated with saliva, blood, and commonly used silicone pastes, which most likely impairs the required bonding performance [9,10,11,12,13,14]. Data on the bonding performance of high translucency zirconia after saliva decontamination procedures is sparse and cannot be compared to silica-based ceramics or Y-TZP because of structural differences. Therefore, the objective of this in vitro study was to evaluate the shear bond strength and fracture mode of three adhesive luting systems on high translucency zirconia surfaces. It was hypothesized that (1) different cleaning and activating procedures of the ceramic surface allow for an adequate bond strength between high translucency zirconia and adhesive luting agents. Furthermore, the authors hypothesized that (2) the preliminary surficial treatment would not impair the bond strength or fracture mode after aging procedures (24 h, TC, or 90 days).

## 2. Results

**Figure 1:**
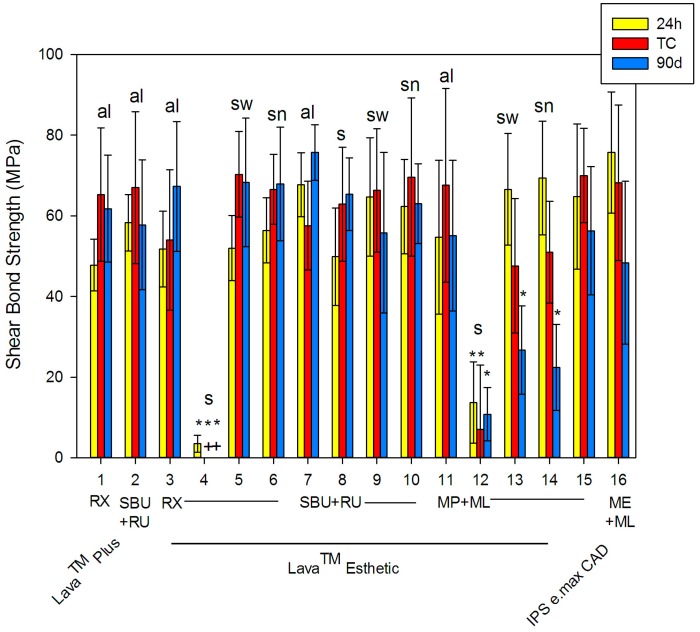
Shear bond strength after 24 h, thermocycling (TC), and 90 days (mean ± standard deviation). Means with * are statistically different compared to non-contaminated specimens (al) of the same adhesive luting system at *p* > 0.05. The + indicates a deficiency in the shear bond strength. Figure abbreviations are as follows: thermocycling (TC), alcohol (al), saliva (s), saliva + water (sw), saliva + water + NaOCl (sn), Scotchbond^TM^ Universal Adhesive (SBU), Monobond Plus (MP), Monobond Etch & Prime (ME), RelyX^TM^ Unicem 2 (RX), RelyX^TM^ Ultimate (RU), and Multilink^®^ Automix (ML).

The shear bond strength (SBS) of high translucency zirconia, standard zirconia, and lithium disilicate ceramic significantly (*p* < 0.001) differed from 3.5 ± 2.1 MPa (#4) to 75.7 ± 15.0 MPa (#14) after 24 h (see Figure 1 and Table 1). Only saliva contamination severely impaired the SBS of two luting systems, resulting in SBS values lower than 13.7 MPa ± 10.1 MPa (#12). Interestingly, Scotchbond^TM^ Universal Adhesive (SBU) + RelyX^TM^ Ultimate (RU) was not statistically significant in contrast to non-saliva-contaminated systems. 

**Figure 2:**
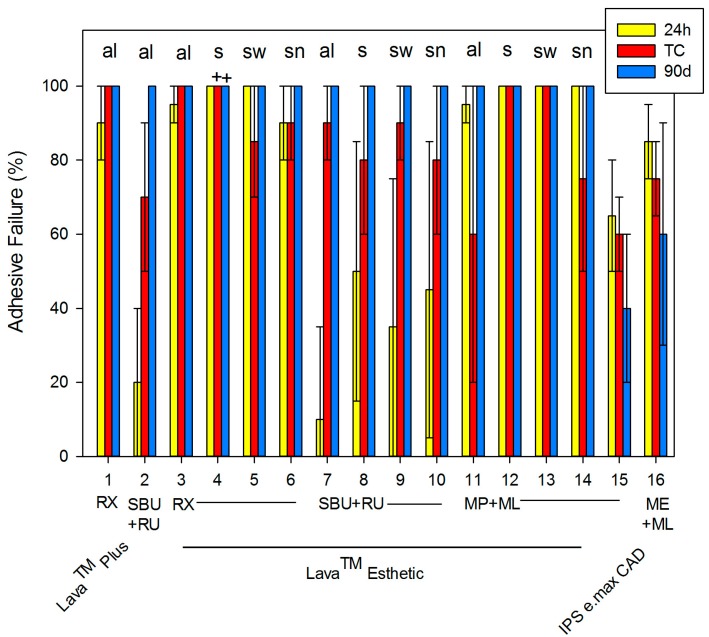
Percentage of adhesive failures after 24 h, thermocycling (TC), and 90 days (mean ± standard deviation). The + indicates a deficiency in the shear bond strength, and the figure abbreviations are as follows: thermocycling (TC), alcohol (al), saliva (s), saliva + water (sw), saliva + water + NaOCl (sn), Scotchbond^TM^ Universal Adhesive (SBU), Monobond Plus (MP), Monobond Etch & Prime (ME), RelyX^TM^ Unicem 2 (RX), RelyX^TM^ Ultimate (RU), and Multilink^®^ Automix (ML).

After thermocycling (TC), the SBS varied significantly (*p* < 0.001) from 0 (#4) to 70.3 ± 10.6 MPa (#5). Again, saliva (s) contamination showed lowermost values between 0 and 7 ± 16.0 MPa for RX (#4) and MP + ML (#12), which were statistically significant in contrast to all other systems. With TC, 11 systems showed an increase in SBS, whereas the SBS of 5 systems decreased. Thus, the temperature difference of TC (5–55 °C) affects the chemical and mechanical bond between adhesive luting systems and ceramic. In comparison to the baseline, most systems showed statistically significant differences. After 90 days of storage, SBS values ranged from 0 (#4) and 10.8 ± 6.6 MPa (#12) due to saliva contamination to 75.7 ± 6.9 MPa (#7). Compared to the baseline, eight systems showed an increase in SBS. Compared to TC, four systems showed an increase in SBS. In general, statistically significant differences were identified between the different groups at each time point (*p* < 0.001). Moreover, adhesive failures were predominantly observed at all aging conditions (see Figure 2).

## 3. Discussion

The hypothesis of this in vitro study is partially confirmed: (1) different cleaning and activating procedures for high translucency zirconia surfaces allow for effective bond to adhesive luting agents for all systems. However, the results of the present study led us to partially reject the hypothesis in that (2) preliminary surficial treatment does not affect the bond strength and fracture mode after aging because there are significant differences in the SBS between different time points. 

The clinical long-term performance of ceramic restorations is related to an appropriate bonding procedure [1,2,3] influenced by many factors, such as contamination [9,10], primer coating [15], surface treatment [16], and air abrasion [17]. In this in vitro study, bonding performance was tested according to ISO/TS 11405:2015 in terms of the SBS of high translucency zirconia to CoCr cylinders after aging procedures (24 h, TC, and 90 days of water storage). Although human or bovine enamel and dentin are often used for SBS testing, CoCr cylinders were used in this in vitro study to focus on the bonding performance of high translucency zirconia and eliminate tooth substance characteristics. Considering that irregular stress distributions at the tested interface, which result in initial failure at the insertion point of the load, are disadvantageous, SBS tests are still comparatively simple and effective in in vitro screening methods of adhesive luting systems prior to more defined in vivo tests [12]. While there is no clear benchmark for maximum or minimum shear bond strengths to date, the bonding of dental restorative materials should resist the mechanical, thermal, and chemical forces of the oral cavity but avoid tooth substance loss after debonding [18,19,20]. The optimal bonding forces of orthodontic materials are theoretically set at 5–50 MPa [18]. The SBS of the high translucency zirconia ranged from 0 to 75.7 MPa, which is high compared to results of other studies testing SBS or µSBS [9,12,15]. Therefore, two commonly used ceramics, a lithium disilicate ceramic (IPS e.max CAD) and a 3Y zirconia (Lava™ Plus), were included here showing similar results (47.8–75.7 MPa) in this experimental set-up. Bond failure of the present study predominately occur at the interface between adhesive luting systems and high translucency zirconia, suggesting that the weakest link was loaded during SBS testing. 

In general, manufacturers recommend specific pre-treatments for the inner ceramic surface before bonding to achieve a sufficient micromechanical and chemical interlocking between ceramic restorations and adhesive luting systems. A recently introduced single-component ceramic primer Monobond Etch & Prime (ME), which includes an alcoholic aqueous solution of ammonium polyfluoride and silane methacrylate, allows for the etching and priming of silica-based ceramic in one step. Limited comparative studies (hydrofluoric acid (HF) + MP vs. ME) [4,5] show conflicting results compared to the present investigation; they reveal equivalent initial bond strength results (24 h), but no information is available after artificial aging. There are no significant differences (SBS and failure mode) between adhesively bonded lithium disilicate ceramic (IPS e.max CAD) using HF + MP and ME in combination with the resin cement ML after TC or 90 days. 

During the intraoral try-in procedure, restoration surfaces come in close contact with the gingiva, causing contamination with saliva, sulcus fluid, and blood. Different cleaning protocols for zirconia and their influence on bond strength have been reported in the literature. These include the application of water, alcohol, phosphoric acid, or universal cleaning paste [2,11]. Some authors have also suggested airborne-particle abrasion as the most efficient method for cleaning zirconia from saliva and ensuring bond durability [14]. However, no information about cleaning procedures for high translucency zirconia was found. Saliva contamination resulted in almost complete bond strength failure in this investigation. Prior bonding all compounds of phosphate, such as phospholipids from saliva or phosphoric acid residues, are considered to be problematic, because this may impair bonding effectiveness [10,12] due to formation of zirconium phosphate. Subsequently, zirconia surfaces are inert to phosphoric-acid-containing reagents [14], such as MP, SBU, and RX. Nevertheless, SBS values of RX are not significantly different in comparison to systems without saliva coating (al). This may be explained by the setting reaction of RX to chemically crosslinking methacrylates and the formation of “methacrylated” phosphates and calcium phosphates. 

Previous studies conflict in their conclusions on saliva-contaminated conventional zirconia surfaces rinsed with water before a light-cured composite resin is applied, because saliva immediately coats surfaces with a proteinaceous layer [21]. On the one hand, water rinsing is less effective on shear bond strength [10], but on the other hand, cleaning with water is as efficient as NaOCl + water or comparable to the SBS of non-contaminated zirconia [9]. Decontamination with water was comparable to NaOCl + water, suggesting that the decontamination with water was sufficient without the physico-chemical properties of NaOCl in the present study. The combination of NaOCl + water for decontamination appears to be highly efficient on bond strength when conventional zirconia is bonded to a self-adhesive resin cement [13]. This is in accordance with the present data on all adhesive luting systems bonded to high translucency zirconia. Coating with 10-MDP (10-methacryloyloxydecyl dihydrogen phosphate) prior to saliva contamination was followed by a water-preserved bond strength [10], suggesting that the zirconia surface is somewhat saturated and that the interaction of phosphate compounds from saliva is impossible. 10-MDP is the functional monomer of SBU, which is a universal adhesive to bond tooth structures and various dental materials. A chemical interaction of 10-MDP and Y-TZP through ionic and hydrogen bonding has been described [22]. Although there are no available reports on the bonding performance between adhesive luting systems that include a universal adhesive, such as SBU, and high translucency zirconia, those adhesives effectively bonded to CAD or CAM zirconia materials [23,24] as seen in the present study. 

A decrease in SBS is usually expected after artificial aging (TC). SBS of MP + ML bonded high translucency zirconia was reduced by TC. When comparing the 24 h results with thermocycling RX or RBU + RX bonded Lava™ Esthetic, an increase in SBS was observed. There was no further increase after 90 days of water storage in any case, but the values were elevated compared to the baseline data. It can be assumed that, besides an initially and primarily micromechanical bond, the functional methacrylates of adhesive luting systems bond chemically to the ceramic. The comparison of SBS of primer (MP) + resin cement (ML) revealed a statistically significant reduction after 90 days, especially for the saliva-contaminated and subsequently cleaned zirconia, indicating the failure of the bond strength through hydrolysis. 

## 4. Materials and Methods

Whole unstimulated saliva was collected after informed consent from a single 52-year-old, healthy female volunteer by expectoration at different occasions and frozen immediately at −30 °C. Immediately prior to the experiments saliva was gently defrosted, pooled, and successively sterilized with single use filtration devices (bottle tip filter/pore size 0.45 µm and 0.22 µm, Corning Inc., Corning, NY, USA). All in all, 360 specimens were fabricated from the high translucency zirconia Lava™ Esthetic (3M Oral Care, Seefeld, Germany) with the following dimensions: thickness: 2 mm; length: 20 mm; width: 10 mm. A lithium disilicate ceramic (IPS e.max CAD; *n* = 60; Ivoclar Vivadent, Schaan, Liechtenstein) and a 3Y zirconia (Lava™ Plus; *n* = 60; 3M Oral Care, Seefeld, Germany) were used as references. The shear surfaces of the ceramic materials were differentially treated prior to bonding. First, zirconia surfaces were sandblasted (40 µm aluminum oxide, 2 bar). To approximate the usual clinical situation surfaces were subsequently treated with 70% alcohol (al), saliva (s), saliva + water (sw), or saliva + 5% NaOCl + water (sn) for 3 min each. The lithium disilicate ceramic was either etched with a 5% hydrofluoric acid gel (Vita Ceramics Etch; Vita Zahnfabrik, Bad Säckingen, Germany) or prepared with Monobond Etch & Prime (ME; Ivoclar Vivadent, Schaan, Liechtenstein). Following surface preparation, the specimens were bonded to CoCr cylinders (diameter 5 mm, height 3 mm) [25] by means of the following adhesive luting systems: RelyX^TM^ Unicem 2 (RX), Scotchbond^TM^ Universal (SBU) + RelyX^TM^ Ultimate (RU), and Monobond Plus (MP) + Multilink^®^ Automix (ML). Overall, 16 different combinations of activation and cleaning procedures were discriminated (Table 1). At a crosshead speed of 1 mm/min, the shear bond strength (SBS) of 10 specimens per testing group was evaluated according to ISO/TS 11405:2015 after 24 h of storage at 37 °C in distilled water (baseline), thermal cycling (TC: 12,000 × 5 °C/55 °C in distilled water), and 90 days of storage at 37 °C in distilled water. For that purpose, all specimens were placed in the shear bond device of a universal testing machine (Zwick 1446; Zwick, Ulm, Germany) in which the loading die struck the CoCr cylinder. The chisel-shaped rod was set at a distance of 0.1 mm from the alloy panel to avoid cantilever effects on the adhesive surface. Failure modes, such as adhesive, cohesive, or mixed fractures, along the high translucency ceramic surface were determined by percentage of bonded area using a reflected light microscope (Stereoscan; Zeiss, Jena, Germany). Means and standard deviations (*n* = 10 per group) were calculated and statistically analyzed with one-way analysis of variance (ANOVA) and a Bonferroni correction (α = 0.05).

## 5. Conclusions

Cleaning and decontamination procedures (alcohol, water, and NaOCl + water) are crucial for obtaining an effective bond between various adhesive luting systems and high translucency zirconia. Only the bond of the universal adhesive SBU, together with the resin cement RU, to zirconia withstood saliva contamination. Within the limitations of the present study, the use of a self-etching cement or adhesive bonding in combination with cleaning and decontamination appears to guarantee efficient bonding to high translucency zirconia. However, further studies are needed to predict the clinical long-term performance of the adhesive bond of formerly contaminated high translucency zirconia.

## Figures and Tables

**Table 1:** Shear bond strength after 24 h, thermocycling (TC), and 90 days (mean ± standard deviation).

#	Ceramic	Pre-Treatment	Procedure	Primer	Cement	Mean (SD)		
						24 h	TC	90 days
1	Lava™ Plus	sandblasting	al	none	RX	47.8 (6.4)	65.3 (16.5)	61.8 (13.3)
2				SBU	RU	58.3 (7.0)	67.0 (18.8)	57.8 (16.1)
3	Lava™ Esthetic		al	none	RX	51.8 (9.4)	54.0 (17.4)	67.3 (16.1)
4			s			3.5 * (2.1)	0 * (0)	0 * (0)
5			sw			52.0 (8.1)	70.3 (10.6)	68.3 (15.9)
6			sn			56.4 (8.1)	66.6 (8.7)	67.9 (14.1)
7			al	SBU	RU	67.7 (7.9)	57.6 (11.0)	75.7 (6.9)
8			s			49.9 (12.1)	62.9 (14.1)	65.4 (9.0)
9			sw			64.7 (14.7)	66.3 (15.3)	55.8 (19.9)
10			sn			62.3 (11.7)	69.6 (19.6)	63.0 (9.9)
11			al	MP	ML	54.7 (19.1)	67.6 (24.0)	55.1 (18.7)
12			s			13.7 * (10.1)	7.0 * (16.0)	10.8 * (6.6)
13			sw			66.6 (13.8)	47.6 (16.7)	26.7 * (11.0)
14			sn			69.4 (14.1)	51.0 (12.6)	22.4 * (10.7)
15	IPS e.max CAD	5% HF	none	MP		64.8 (18.0)	70.0 (11.7)	56.3 (15.9)
16		none		ME		75.7 (15.0)	68.2 (19.3)	48.4 (20.2)

Means with * are statistically different compared to non-contaminated specimens (al) of the same adhesive luting system at *p* < 0.05. The table abbreviations are as follows: standard deviation (SD), thermocycling (TC), hydrofluoric acid (HF), alcohol (al), saliva (s), saliva + water (sw), saliva + water + NaOCl (sn), Scotchbond^TM^ Universal Adhesive (SBU), Monobond Plus (MP), Monobond Etch&Prime (ME), RelyX^TM^ Unicem 2 (RX) (3M Oral Care, Seefeld, Germany), RelyX^TM^ Ultimate (RU) (3M Oral Care, Seefeld, Germany), and Multilink^®^ Automix (ML) (Ivoclar Vivadent, Schaan, Liechtenstein).
